# Immunopathology of Postprimary Tuberculosis: Increased T-Regulatory Cells and DEC-205-Positive Foamy Macrophages in Cavitary Lesions

**DOI:** 10.1155/2011/307631

**Published:** 2010-12-21

**Authors:** Kerry J. Welsh, Semyon A. Risin, Jeffrey K. Actor, Robert L. Hunter

**Affiliations:** Department of Pathology and Laboratory Medicine, University of Texas Medical School at Houston, 6431 Fannin, MSB 2.136, Houston, TX 77030, USA

## Abstract

Postprimary tuberculosis occurs in immunocompetent people infected with *Mycobacterium tuberculosis*. It is restricted to the lung and accounts for 80% of cases and nearly 100% of transmission. Little is known about the immunopathology of postprimary tuberculosis due to limited availability of specimens. Tissues from 30 autopsy cases of pulmonary tuberculosis were located. Sections of characteristic lesions of caseating granulomas, lipid pneumonia, and cavitary stages of postprimary disease were selected for immunohistochemical studies of macrophages, lymphocytes, endothelial cells, and mycobacterial antigens. A higher percentage of cells in lipid pneumonia (36.1%) and cavitary lesions (27.8%) were positive for the dendritic cell marker DEC-205, compared to granulomas (9.0%, *P* < .05). Cavities contained significantly more T-regulatory cells (14.8%) than found in lipid pneumonia (5.2%) or granulomas (4.8%). Distribution of the immune cell types may contribute to the inability of the immune system to eradicate tuberculosis.

## 1. Introduction

*Mycobacterium tuberculosis* (MTB) is endemic in every part of the world. This organism accounts for nearly 2 million deaths annually, making it the leading bacterial cause of death worldwide [1]. Once thought to be controlled, tuberculosis (TB) incidence is rising in many areas caused in part by the emergence of drug-resistant strains and the HIV epidemic. Furthermore, nearly one-third of the world is latently infected with MTB, making eradication of the organism difficult. 

MTB infection begins as primary TB with the deposition of bacilli in the alveoli, which are phagocytosed by alveolar macrophages [2]. Numerous animal models of primary TB are available and much is known about the immune responses that drive granuloma development [3]. Macrophage production of TNF-**α** and chemokines recruits systemic monocytes which form the nascent granuloma. Adaptive immunity develops after infected dendritic cells (DCs) migrate to a draining lymph node [4, 5]. Neutrophils may also facilitate the presentation and migration of mycobacterial antigens to draining lymph nodes [6]. IL-12 production by DCs promotes the development of IFN-*γ* Th1 cells that activate macrophages, thereby stabilizing bacterial growth [3]. The development of an acquired immune response results in the formation of a mature caseating granuloma that consists of central necrosis and infected macrophages surrounded by epitheliod and foamy macrophages and an outer layer of fibrosis associated with lymphocytes [7]. Most of such lesions heal, but viable organisms persist. Patients at this stage of infection are considered latently infected because they lack clinical symptoms and have stable chest X-rays, and sputums are negative for acid-fast bacilli [8]. The TB bacilli persist, despite the strong immune response needed to generate granulomas. This is possibly explained by the finding that the acquired immune response to MTB is delayed in comparison to other infectious diseases [9]. 

A fraction of latently infected individuals develop reactivation of infection or are reinfected from the environment [10]. Such infection of a previously immune or sensitized person produces a very different type of disease known as postprimary TB (also referred to as adult type or secondary infection). Postprimary TB is typically restricted to the lung where it produces cavities that are responsible for 80% of all TB disease and virtually 100% of transmission of the bacilli from person to person [11]. Postprimary TB occurs characteristically in young immunocompetent adults with sufficient immunity to control primary TB [12]. 

 It is a paradigm of contemporary research that caseating granulomas are the characteristic lesion of both primary and postprimary TB and that cavities form by expansion of caseating granulomas with softening and liquefaction of their contents that are discharged as they erode into bronchi. We previously reported studies of untreated primary and postprimary TB that contradicted this paradigm [13]. Based on our observations from preantibiotic era derived samples, we hypothesized that postprimary TB begins as a lipid pneumonia in which infection is restricted to foamy alveolar macrophages. Therefore, resultant cavities appeared to result from necrosis of tuberculous pneumonia in individuals who had no histologic evidence of caseating granulomas. The present studies were undertaken to characterize the cellular makeup of each of the characteristic stages of human pulmonary TB in immunocompetent adults. We examined slides of the lungs of 30 people who died of pulmonary TB and selected representative sections of each of the major stages of the disease. These were (1) caseating granulomas of primary TB, (2) lipid pneumonia, and (3) cavitary lesions. These sections were studied with immunohistochemical markers of T-cell subsets, macrophages, DCs, and endothelial cells based on published studies of these markers in animal models of TB. Immunohistochemical analysis of MTB antigens and acid fast bacilli (AFB) staining was included to explore our hypothesis that a progressive buildup of mycobacterial antigens precedes and contributes to the rapid necrosis of caseous pneumonia [11]. These studies were accomplished with a multispectral imaging microscope and image analysis software that facilitates accurate differentiation and quantitation of multiple cell types with precise quantitation of immunohistochemical markers. 

The results demonstrate distinct patterns of immune cells in each of the stages of pulmonary TB. Foamy macrophages (FMs) found in lipid pneumonia and in the walls of cavitary lesions were frequently positive for the DC marker DEC-205. An increased percentage of T-regulatory (Treg) cells in the cavity walls of TB provide evidence of an active role in the disease process. It is hypothesized that the local accumulation of immune cells with suppressive properties may contribute to the chronic nature of MTB infection.

## 2. Methods

### 2.1. Tissue Specimens

Formalin-fixed, paraffin-embedded tissue blocks from patients with postprimary TB were obtained from the First Infectious Disease Hospital, St. Petersburg, Russian Federation. Specimens were from archived cases of autopsies of patients who had died of pulmonary TB and were culture positive for MTB or had positive stains for acid-fast bacilli. Thirty cases of postprimary TB patients were available for investigation. Although detailed information is not available, all patients were HIV negative. All cases were stained with hematoxylin and eosin and acid fast stained by the Ziehl-Neelsen method per standard protocols. 

This study was conducted according to the principles expressed in the Declaration of Helsinki. The study was approved by the Institutional Review Board of UT-Houston Medical School IRB protocol number HSC-MS-10-0109, Immunopathology of Tuberculosis. We studied microscope slides of tissues from patients with tuberculosis. All materials were byproducts of regular autopsy practice and were obtained after completion of all medical needs. The specimens were deidentified for the study.

### 2.2. Immunohistochemistry Staining for Immune Cell Markers

Five-**μ**m tissue sections were deparaffinized and stained with monoclonal antibodies for CD4 (Leica-Microsystems, Bannockburn, IL), CD8 (Dako, Carpinteria, CA), CD20 (Dako), CD31 (Leica-Microsystems), CD68 (Dako), DEC-205 (Santa Cruz Biotechnology, Santa Cruz, CA), and Foxp3 (Abcam, Cambridge, MA). Mouse monoclonal antibodies were used for all immune cell markers. A rabbit polyclonal antibody to purified protein derivative (Abcam) was used for the detection of MTB. The pretreatments, dilutions, and incubation times are shown in Table 1. After washing, sections were incubated for one hour with antimouse (Dako) or antirabbit (Biocare Medical, Concord, CA) polyclonal antibodies labeled with HRP. 

### 2.3. Photography and Image Analysis

All images were taken using the Nuance multispectral imaging system (CRI, Woburn, MA), which allowed enumeration of cellular phenotypes in defined areas of pathology. Three distinct pathologic manifestations of TB were analyzed that included granulomas, lipid pneumonia, and cavitary lesions. Images from at least 5 cases of the defined pathological manifestations were captured with the 10x objective. Quantification of immune cells expressing specific markers was performed with the tissue and cell segmenting functions of Inform software (CRI), according to the manufacturer's instructions. The total number of cells in an image was determined using the hematoxylin stain.

## 3. Results

### 3.1. Granulomatous Pathology

**Figure 1:**
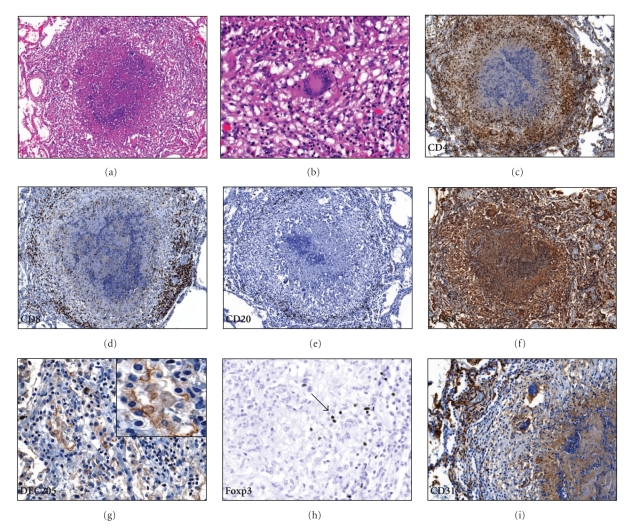
Granuloma histopathology. (a) Typical caseating granuloma, with central necrosis flanked by epitheliod cells and foamy macrophages. The periphery of the granuloma consists of lymphocytes, 100x. (b) Langhan's giant cell, 400x. (c) Abundant CD4+ cells in the granuloma include both lymphocytes and macrophages, 100x. (d) CD8+ cells were mainly located in the periphery of the granuloma, 100x. (e) B-cells, identified by the CD20 stain, were noted in the outer layer of the granuloma. (f) Granulomas stained heavily for the CD68 marker, 100x. (g) Macrophages at the periphery of the granuloma stain positive for the dendritic cell marker DEC-205, 400x. The insert demonstrates a digitally zoomed region to detail the DEC-205 staining. (h) T-regulatory cells, identified by the Foxp3 marker (arrow), were found in the lymphocytic layer of the granuloma, 400x. (i) CD31 stain shows well-vascularized tissue at the periphery of the granuloma, 200x.

Three distinct patterns of pathology of pulmonary TB were analyzed. The first type is the caseating granuloma characteristic of primary TB. Similar caseating granulomas were observed in primary TB, miliary TB, and chronic fibrocaseous postprimary TB. A representative image is shown in Figure 1(a). A central region of caseous necrosis is surrounded by foamy and epitheliod macrophages, often presenting with Langhan's giant cells (Figure 1(b)). The percentages of different immune cell markers obtained by quantitative image analysis are shown in Table 2. Granulomas contained an abundance of CD68+ macrophages and CD4+ lymphocytes (Figures 1(f) and 1(c)). CD8+ lymphocytes, as well as B-cells identified by the CD20 marker, were found in the lymphocytic outer region of the granuloma (Figures 1(d) and 1(e)). The outermost layer of the granuloma near the alveoli consisted of FMs that were frequently positive for the DC marker DEC-205 (Figure 1(g)). Foxp3, a marker for Treg cells, were occasionally noted interspersed between other stained lymphocytes (Figure 1(h)). The periphery of granulomas was well-vascularized as assessed by the CD31 stain (Figure 1(i)).

### 3.2. Lipid Pneumonia

**Figure 2:**
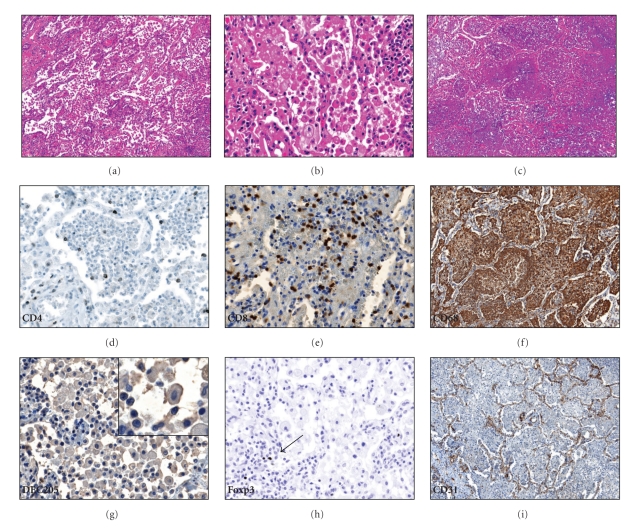
Lipid pneumonia. (a) Alveoli are filled with macrophages and the alveolar walls have high numbers of lymphocytes, 100x. (b) Macrophages in the lipid pneumonia have a foamy appearance, 400x. (c) The cells in the alveoli may undergo necrosis to produce a homogenous appearance, 100x. (d) Relatively few CD4+ cells are present in lipid pneumonia, 400x. (e) Abundant CD8+ cells are noted in the alveoli as well as in the alveolar walls, 400x. (f) Foamy cells stain strongly with the CD68 marker. (g) Foamy macrophages in the lipid pneumonia are frequently positive for the dendritic cell marker DEC-205, 400x. The insert shows a detailed image of a DEC-205-positive macrophage. (h) T-regulatory cells were observed in regions of lipid pneumonia (arrow), 400x. (i) The CD31 stain highlights the disrupted lung architecture, 100x.

The alveoli were filled with FMs positive for the CD68 marker with lymphocytes present in the alveolar walls (Figures 2(a)–2(c)). Cells in the alveoli occasionally had a “dry” appearance with areas of fibrosis (Figure 2(a)) or a homogenous consistency of necrotic material (Figure 2(c)). Bronchial obstruction was frequently present [13]. CD4+ cells were present at significantly less frequency compared to numbers seen in granulomas (*P* < .05, Table 2 and Figure 2(d)). CD8+ cells were frequently observed in the alveolar walls as well as the alveoli (Figure 2(e)). B-cells were rarely noted in the regions of lipid pneumonia but were observed to occur in large clusters in regions that bordered the lipid pneumonia (not shown). The FMs in the alveoli were frequently positive for the DC marker DEC-205 (Figure 2(g)). Treg cells were noted in the alveoli but were scarce elsewhere (Figure 2(h)). Disrupted lung architecture of developing caseaous necrosis was readily apparent on the CD31 stain of vascular endothelial cells (Figure 2(i)).

### 3.3. Cavitary Lesions

**Figure 3:**
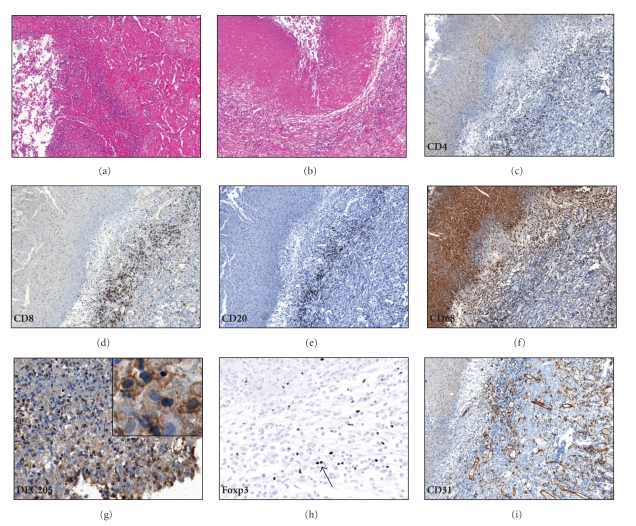
Immunopathology of cavitary lesions. (a) Early cavitary lesion, surrounded by lipid pneumonia, 100x. (b) Chronic cavitary lesion. The cavity wall consists of necrotic material, foamy macrophages, and fibrous tissue with abundant lymphocytes, 100x. (c) Numerous CD4+ cells make up the lymphocytic portion of a cavity wall, 100x. (d) CD8+ cells in the wall of a cavity, 100x. (e) CD20+ cells in the wall of a cavitary lesion, 100x. (f) The cells lining the cavity are CD68+ macrophages, 100x. (g) Foamy macrophages lining the cavity wall are positive for the dendritic cell marker DEC-205, 400x. Insert demonstrates a region detailing the staining for DEC-205. (h) Numerous Foxp3+ cells (arrow) were found in the cavity wall, 400x. (i) Abundant granulation tissue in the wall of a cavitary lesion noted with the CD31 stain, 100x.

An example of a cavity that appears to be developing by necrosis and fragmentation of lipid pneumonia is shown in Figure 3(a). The cavity wall is made up of necrotic debris, foamy macrophages, and lymphocytes. Abundant fibrosis develops as the lesion becomes chronic (Figure 3(b)). The lymphocytes lining the walls of mature cavities were a mixture of CD4, CD8, and CD20 positive cells (Table 2 and Figures 3(c)–3(e)). The FMs lining the cavity wall were strongly positive for the DC marker DEC-205 (Figure 3(g)). Numerous Treg cells were present around the cavities (Figure 3(h)). The walls of the cavitary lesions contained abundant granulation tissue (Figure 3(i)). None of the cavitary lesions were directly associated with caseous granulomatous pathology.

### 3.4. Localization of MTB Antigens and Acid-Fast Bacilli

**Figure 4:**
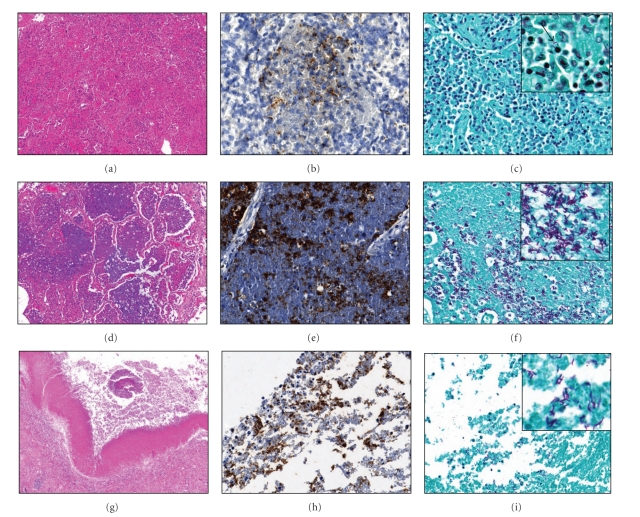
Comparison of immunohistochemistry (IHC) for MTB and Zhiel-Neelsen acid fast staining. Early lipid pneumonia (a)–(c). (a) H&E stain demonstrates macrophages and lymphocytes within the alveoli, 100x. (b) MTB are noted on the IHC, 400x. (c) Few AFB are observed by Zhiel-Neelsen staining, 400x. Lipid pneumonia undergoing abrupt necrosis (d)–(f). (d) Cells in alveoli undergo necrosis and have a homogenous appearance, 100x. (e) Abundant mycobacterial antigens noted on the IHC stain for MTB, 400x. (f) Numerous acid-fast bacilli are observed, 400x. Cavitary TB (g)–(i). (g) H&E stain showing a cavity with a piece of detached lung and necrotic debris, 40x. (h) The wall of the cavity is strongly positive for MTB antigens, 400x. (i) Acid-fast bacilli are found lining the cavity wall, 400x.

MTB were rarely observed in granulomas by either Zhiel-Neelsen staining or immunohistochemistry for MTB. Representative images from cases of lipid pneumonia and cavitary lesions are shown in Figure 4. Early lipid pneumonia demonstrated evidence of mycobacterial antigens throughout alveoli, but very few acid-fast bacilli were seen (Figures 4(a)–4(c)). As the lipid pneumonia undergoes necrosis, both abundant tuberculin antigens and bacilli are evident (Figures 4(d)–4(f)). Cavities had roughly equal amounts of antigen and bacteria, most of which were localized to regions representing the cavity wall (Figures 4(g)–4(i)).

## 4. Discussion

A central question in TB research is why the immune system successfully controls primary TB but fails to clear a postprimary TB cavity and thus halt transmission of infection. A problem is that investigators have little to study since few specimens of human pulmonary TB are available to them. Recent descriptions of specimens recovered from surgical cases of patients with cavitary TB are available [14]. This project was built on earlier studies that described the histopathology of the stages of postprimary TB [13]. To our knowledge, this study is the first recent description of postprimary TB that includes Tregs and cells with the DEC-205 marker.

Histopathologic examination of the 30 cases in this study supported our previous hypothesis that postprimary TB begins as a lipid pneumonia [13]. Other authors suggest that cavities develop when granulomas rupture into the airways and that lipid pneumonia is a complication of cavitary TB [2, 3, 15]. Analysis of the development of cavities is beyond the scope of this paper. Nevertheless, a key question of postprimary TB is how MTB survive and proliferate in alveolar macrophages in restricted areas of lung while the entire rest of the body remains highly immune. The present studies provide some clues. Foamy macrophages positive for the DC marker DEC-205 accumulate in the alveoli with CD8+ lymphocytes in the alveolar walls and alveoli. Ordway and colleagues [16] reported that FMs from murine granulomas were strongly positive for the DC marker DEC-205. Interestingly, a high percentage of FMs found in lipid pneumonia and those lining the cavity walls were positive for DEC-205. The FMs in mice were reported to have additional markers of DCs such as CD11c, MHC class II, and CD40 as well as high levels of antiapoptotic markers [16]. The significance of FMs expressing DC markers is not yet clear. However, MTB-infected DCs can harbor the bacilli for extended periods of time and do not have efficient mechanisms of eliminating MTB [17–19]. FMs with characteristics of DCs may therefore provide a permissive environment for the growth of mycobacteria. Furthermore, FMs may be a tissue source of immunosuppression that inhibits the cell-mediated immunity necessary to clear MTB. For example, FMs secrete high levels of transforming growth factor-beta (TGF-*β*) that can cause apoptosis of immune effector cells [20]. Additionally, FMs produce high levels of inducible nitric oxide synthetase, which has been associated with suppression of T-cells in murine MTB infection [21]. Therefore, FMs may be a contributor to the local immunosuppression responsible for the inability of the immune system to eliminate postprimary TB. 

Organisms in cavities typically grow in massive numbers on the inner surface of a cavity but nowhere else in the body even though other parts of the body, particularly the lung, are continually exposed to large numbers of virulent organisms being coughed into the environment. The surface of a cavity has been described as an area of “failed immunity” [22]. This study found a significantly increased percentage of Treg cells in the cavitary wall compared to other tissue manifestations of TB. Treg cells, identified by expression of the transcription factor Foxp3, are essential for preventing self-reactive immune responses and limiting immune-mediated tissue damage during infection [23, 24]. However, the role of Tregs in infectious diseases such as TB is complex. Tregs produce inhibitory cytokines, such as IL-10 and TGF-*β*, that protect host tissue by limiting excess inflammation but may conversely limit clearance of pathogens [25]. Peripheral blood Treg cells are decreased in newly infected contacts of TB patients, possibly due to accumulation in infected lung [26]. As the infection progresses, Tregs expand to regions of disease sites, as well as in blood [27–30]. Differential expression of Foxp3 in PBMCs was predictive of active TB versus latent infection [31]. Of significance, Foxp3 cells decreased T-cell-mediated responses to mycobacterial antigens in human TB [32]. Depletion of Treg cells during a murine model of infection enhanced MTB elimination [33, 34], providing further evidence that Treg cells inhibit the clearance of MTB. A recent study demonstrated that small numbers of MTB-specific Tregs inhibit the accumulation of CD4 and CD8 T-cells in the lungs of infected mice [35]. The findings from our study demonstrate high numbers of Treg cells in a tissue manifestation of TB from which the bacilli are frequently not eradicated, providing further support to the hypothesis that accumulation of Tregs at the sites of MTB infection suppresses the activation of protective immune responses. However, it should be noted that Treg activity was not the limiting factor in the effectiveness of BCG vaccination of mice [36]. However, since mice do not develop cavities, the interpretation of such studies for the human host response to cavities must be taken with caution.

These studies also confirmed earlier observations that large amounts of antigens of MTB demonstrable by immunohistochemistry are present in alveoli of the lipid pneumonia [13]. In this stage, there was far more MTB antigen in alveoli than organisms observed by acid-fast staining. Much of it was present in structures that were distinct from aggregates of AFB. We hypothesize that mycobacterial antigens accumulate progressively in alveoli during this stage of disease. This is similar to the accumulation of MTB antigens in alveoli of slowly progressive pulmonary TB in mice [13]. We have proposed that this build up of mycobacterial antigens including glycolipids is an essential precursor to the abrupt necrosis that produces cavities [7, 37, 38]. 

This study has a number of important limitations. Tissues from autopsy cases of patients who died of pulmonary TB were received as deidentified paraffin-embedded blocks. While we were told that the tissues came from untreated or inadequately treated patients, detailed clinical information on a case-by-case basis is not available. Nonetheless, this study provides important details of the cellular phenotypes in characteristic tissue manifestations of postprimary TB.

## 5. Conclusions

In summary, better understanding of the pathology of the multiple stages of postprimary disease has facilitated investigations of manifestations of the disease that have heretofore been unapproachable by modern science. The key questions are how does MTB establish and survive in two privileged sites in the upper lobes of lungs of hosts who are strongly immune in all other parts of the body. The first site is an endogenous lipid pneumonia in which MTB survive only in foamy alveolar macrophages in parts of the lung. The second is a mature cavity in which MTB proliferate in massive numbers on the cavitary surface, but nowhere else. The present studies demonstrate that these questions can be approached with modern quantitative immunohistochemical technologies and that different host responses are operative at each stage of the disease. The results demonstrate that both types of privileged sites are characterized by an increased percentage of cell phenotypes with immunosuppressive properties that may be a critical component responsible for the inability of the immune system to clear MTB. It is anticipated that a better understanding of the immunopathology of MTB, and the molecular mechanisms governing the transition from latent to active disease will ultimately lead to more effective control of TB. 

## Figures and Tables

**Table 1:** Conditions for immunohistochemistry staining of immune cell markers and MTB.

Marker	Pretreatment	Dilution	Incubation time^a^
CD4	steam for 20 minutes in EDTA, pH 8.0	1 : 25	45
CD8	steam for 20 minutes in EDTA, pH 8.0	1 : 125	30
CD20	5 minutes under pressure in citrate buffer, pH 6.0	1 : 800	20
CD31	5 minutes under pressure in citrate buffer, pH 6.0	1 : 100	30
CD68	5 minutes under pressure in citrate buffer, pH 6.0	1 : 2000	20
DEC-205	steam for 20 minutes in EDTA, pH 8.0	1 : 50	overnight
Foxp3	5 minutes under pressure in citrate buffer, pH 6.0	1 : 100	30
MTB	Proteinase K	1 : 100	60

^a^minutes.

**Table 2:** Percentage of cells positive for immunological markers.

	Primary TB	Postprimary TB	Postprimary TB
	Granuloma	Lipid pneumonia	Cavitary lesion
Marker			
CD4	30.7 ± 4.1	10.1 ± 3.1^a^	23.1 ± 4.6^a,b^
CD8	17.8 ± 4.2	19.6 ± 3.8	27.7 ± 7.1
CD20	2.0 ± 0.5	2.3 ± 0.7	7.8 ± 1.2^a,b^
CD68	49.4 ± 5.0	64.0 ± 12.5	55.1 ± 14.8
DEC-205	9.0 ± 3.5	36.1 ± 8.5^a^	27.8 ± 8.5^a^
Foxp3	4.8 ± 1.1	5.2 ± 1.2	14.8 ± 4.3^a,b^

Images of the different tissue manifestation of TB were taken using the 10x objective from at least 5 regions of multiple cases. The total number of cells was determined using the hematoxylin stain. The data are presented as the percentage of cells positive for a given marker, ±SD.

^a^ *P* <  .05, comparison to granuloma.

^b^ *P* <  .05, comparison to lipid pneumonia.
